# Preoperative risk factors for early recurrence and relapse-free survival after R0 resection of gallbladder cancer

**DOI:** 10.1097/MD.0000000000045648

**Published:** 2025-10-31

**Authors:** Sang Hun Lee, Dong Uk Kim, Young Mi Seol

**Affiliations:** a Department of Internal Medicine, Pusan National University Hospital, Pusan National University School of Medicine, Busan, Republic of Korea.

**Keywords:** event-free survival, gallbladder neoplasms, HbA1c, recurrence

## Abstract

Gallbladder (GB) cancer is highly prevalent and fatal in South Korea. In operable patients, radical resection with an R0 margin combined with lymphadenectomy is the mainstay of curative-intent therapy. Despite R0 resection, a high recurrence rate has been observed, necessitating the identification of risk factors for recurrence after surgery. This study aimed to identify risk factors strongly related to recurrence after R0 resection of GB cancer. This single-center, retrospective cohort study included 148 patients with GB cancer who underwent R0 resection between January 1, 2014, and December 31, 2019. Variables were analyzed using statistical tools to identify risk factors related to prognosis. Early recurrence, disease progression within 1 year after R0 resection, relapse-free survival, and time from treatment to any event were analyzed with logistic regression and Cox regression analyses. Early recurrence was observed in 15.5% (N = 23) of patients. Based on statistical analysis, the independent risk factors for early recurrence and relapse-free survival were age > 65 years, glycated hemoglobin level > 6.5%, surgical T stage > T3, surgical N stage > N1, moderate or poor pathological differentiation, reversed albumin–globulin ratio, high C-reactive protein level, and high carbohydrate antigen 19-9 level. Advanced pathological stage and high inflammatory marker levels, reflecting high tumor burden, are associated with poor prognosis. High glycated hemoglobin levels were also associated with early recurrence. In conclusion, active screening for early detection, reduction of inflammatory conditions, and management of diabetes may reduce early recurrence after R0 resection of GB cancer.

## 1. Introduction

Biliary tract cancer is a rare cancer worldwide^[1]^ but shows high prevalence in East Asia, including South Korea.^[2]^ It is classified based on its anatomical location into intrahepatic, perihilar, extrahepatic, and gallbladder (GB) cancers.^[3]^ In non-metastatic disease, radical resection with an R0 margin combined with lymphadenectomy is the mainstay of curative-intent therapy, regardless of the subtype.^[4]^ Despite R0 resection, a high recurrence rate and distant metastasis are frequently observed in biliary tract cancer, necessitating the identification of risk factors related to recurrence.^[5]^

Risk factors for biliary tract cancer include primary hepatobiliary disease (e.g., primary sclerosing cholangitis, cholelithiasis, or viral hepatitis), toxin exposure (e.g., smoking or alcohol use), infections (e.g., *Clonorchis sinensis*), and metabolic factors.^[6]^ Presumably, geographical variations in biliary cancer are related to the different categories of risk factors.^[7]^ Moreover, certain risk factors are correlated with certain anatomical subtypes. Intrahepatic cholangiocarcinoma associated with metabolic factors is more prevalent in Western countries,^[8]^ whereas extrahepatic cholangiocarcinoma resulting from parasitic infections is predominant in Southeast Asia.^[9]^

GB cancer has comparable epidemiologic factors to extrahepatic cholangiocarcinoma,^[10]^ and R0 margin resection is the strongest precondition for curative-intent treatment.^[11]^ If recurrence is experienced despite R0 resection, predisposing risk factors might have a role in progression. This retrospective study attempted to identify risk factors strongly related to recurrence after R0 resection of GB cancer.

## 2. Materials and methods

### 2.1. Patient selection

**Figure 1.**
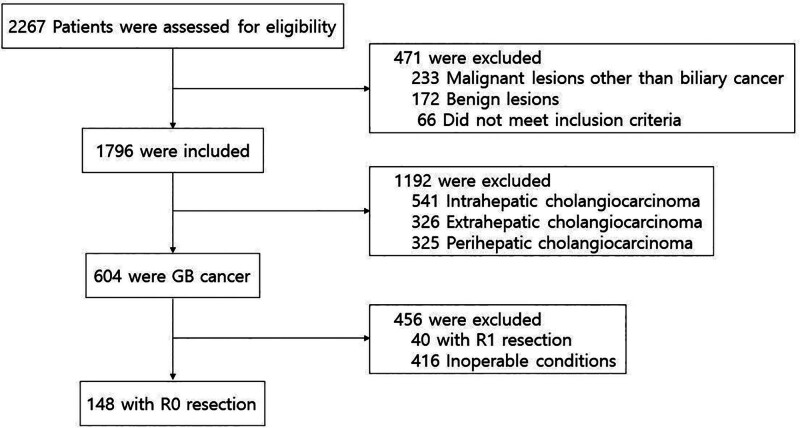
Patient selection scheme. GB = gallbladder.

This single-center, retrospective cohort study included 148 patients with GB cancer who underwent R0 resection. Between January 1, 2014, and December 31, 2019, 2267 patients referred to Pusan National University Hospital because of hepatic or perihepatic masses were assigned. The documentation was based on imaging findings on CT scans or US findings performed at our center or other institutes. After assessments, 471 patients were excluded if their findings suggested other than biliary tract cancer. Of the 1796 patients included, most cases were suggestive of GB cancer. Other biliary tract cancers were intrahepatic, extrahepatic, and perihepatic cholangiocarcinomas, in numeric order. Among the 604 patients diagnosed with GB cancer, 148 underwent R0 resection (Fig. 1). This study was approved by the institutional review board of Pusan National University Hospital (IRB Number 2009-006-094).

### 2.2. Surveillance and monitoring

All enrolled patients were assessed by a history inspection, laboratory parameters, and systemic imaging before surgery. All patients who underwent R0 resection were monitored at 3-to-6-month intervals with routine tests, including liver function tests, tumor markers, and CT. Progression was screened for new lesions detected on the CT images and evaluated according to the Revised International Working Group’s Response Criteria. Early recurrence was defined as disease progression within 1 year after R0 resection. Relapse-free survival (RFS) was defined as the time from the treatment of disease to progression.

### 2.3. Statistical analysis

Relevant variables were analyzed using statistical tools to identify risk factors related to prognosis. Variables were summarized by frequency and percentage for categorical data and mean ± standard deviation and median (range) for numeric data. Group differences were tested using the chi-squared test or Fisher exact test for categorical data and the independent *t* test or Mann–Whitney *U* test for numerical data, as appropriate. To check if the distribution of the variable was normal, we used the Shapiro–Wilk test.

Univariate and multivariate analyses using binary logistic regression were performed to identify prognostic factors that were independently related to early recurrence.

The Kaplan–Meier method was used to estimate RFS and the log-rank test was used to compare survival between the groups. Univariate and multivariate analyses using Cox proportional hazards regression were performed to identify the prognostic factors that were independently related to RFS.

All statistical analyses were performed using SPSS 26.0 (IBM Corp., Released 2019, IBM SPSS Statistics for Windows, Version 26.0. Armonk: IBM Corp), R 4.1.2 (R Core Team (2021). R: Language and environment for statistical computing. R Foundation for Statistical Computing, Vienna, Austria. URL https://www.R-project.org/) and MedCalc Statistical Software version 19.2.6 (MedCalc Software Ltd., Ostend, Belgium; https://www.medcalc.org; 2020). A *P*-value < .05 was considered statistically significant.

## 3. Results

### 3.1. Patients’ characteristics

A total of 148 patients with GB cancer who underwent R0 resection between January 1, 2014, and December 31, 2019, were enrolled. Their median age was 69.5 years (range: 60.0–75.0), and the patients comprised 56 men and 92 women. Demographics, including surgical T and N stages, pathology, metabolic and inflammatory risk factors, and laboratory results, were investigated. Early recurrence was observed in 15.5% (N = 23) of patients (Table 1).

**Table 1:** Patients’ clinical and baseline characteristics (N = 148).

Variable	Overall	GB cancer		
		Early recurrence	Non-early recurrence	*P*
All patients	148 (100.0)	23 (9.1)	125 (49.2)	
Age				
Mean ± SD	66.98 ± 10.86	70.65 ± 8.27	66.30 ± 11.17	.082*
Median (range)	69.5 (60.0–75.0)	71.0 (52.0–85.0)	68.0 (35.0–88.0)	
< 65 yr	53 (35.8)	**3 (13.0**)	**50 (40.0**)	**.013** †
≥ 65 yr	95 (64.2)	**20 (87.0**)	**75 (60.0**)	
Sex				
Male	56 (37.8)	8 (34.8)	48 (38.4)	.742†
Female	92 (62.2)	15 (65.2)	77 (61.6)	
BMI (kg/m^2^)				
Mean ± SD	23.57 ± 3.30	22.59 ± 3.15	23.75 ± 3.31	.138*
Median (range)	23.3 (21.4–25.5)	22.2 (17.6–28.7)	23.4 (16.0–37.2)	
<25.0	106 (71.6)	17 (73.9)	89 (71.2)	.791†
≥25.0	42 (28.4)	6 (26.1)	36 (28.8)	
Surgical T stage				
T1 + T2	126 (85.1)	**10 (43.5**)	**116 (92.8**)	**<.001** ‡
T3 + T4	22 (14.9)	**13 (56.5**)	**9 (7.2**)	
Surgical N stage				
N0	120 (81.1)	**12 (52.2**)	**108 (86.4**)	**.001** ‡
N1 + N2	28 (18.9)	**11 (47.8**)	**17 (13.6**)	
Metabolic factors based on medical records				
HT				
Yes	64 (43.2)	6 (26.1)	58 (46.4)	.071†
No	84 (56.8)	17 (73.9)	67 (53.6)	
DM				
Yes	26 (17.6)	4 (17.4)	22 (17.6)	1.000‡
No	122 (82.4)	19 (82.6)	103 (82.4)	
Dyslipidemia				
Yes	16 (10.8)	3 (13.0)	13 (10.4)	.716‡
No	132 (89.2)	20 (87.0)	112 (89.6)	
Inflammatory factors based on medical records				
Viral hepatitis				
Yes	7 (4.7)	0 (0.0)	7 (5.6)	.596‡
No	141 (95.3)	23 (100.0)	118 (94.4)	
Liver cirrhosis				
Yes	10 (6.8)	1 (4.3)	9 (7.2)	1.000‡
No	138 (93.2)	22 (95.7)	116 (92.8)	
Biliary inflammation				
Cholecystolithiasis + Cholecystitis	59 (39.9)	5 (21.7)	54 (43.2)	.053†
No	89 (60.1)	18 (78.3)	71 (56.8)	
Smoking				
Yes	19 (12.8)	3 (13.0)	16 (12.8)	1.000‡
No	129 (87.2)	20 (87.0)	109 (87.2)	
Alcohol				
Yes	31 (20.9)	4 (17.4)	27 (21.6)	.785‡
No	117 (79.1)	19 (82.6)	98 (78.4)	
Cholesterol				
Mean ± SD	165.65 ± 48.04	154.64 ± 43.45	167.63 ± 48.72	.242*
< 200.0	119 (82.6)	18 (81.8)	101 (82.8)	1.000‡
≥ 200.0	25 (17.4)	4 (18.2)	21 (17.2)	
HbA1C				
Mean ± SD	6.36 ± 1.23	**7.76 ± 2.02**	**6.14 ± 0.90**	**.004** *
<6.5	59 (66.3)	**4 (33.3**)	**55 (71.4**)	**.018** ‡
≥6.5	30 (33.7)	**8 (66.7**)	**22 (28.6**)	
WBC	9.11 ± 4.38	8.98 ± 4.23	9.14 ± 4.43	.970*
Neutrophil (%)	69.78 ± 16.42	68.91 ± 17.52	69.94 ± 16.28	.785*
Lymphocyte (%)	20.30 ± 12.25	21.30 ± 14.05	20.12 ± 11.95	.845*
Neutrophil-to-lymphocyte ratio				
Mean ± SD	6.22 ± 5.84	5.94 ± 5.12	6.27 ± 5.98	.843*
<3.0	61 (41.2)	9 (39.1)	52 (41.6)	.825†
≥3.0	87 (58.8)	14 (60.9)	73 (58.4)	
AST	64.96 ± 105.74	72.09 ± 56.82	63.65 ± 112.56	.343*
ALT	56.39 ± 67.45	62.22 ± 56.81	55.31 ± 69.38	.593*
De Ritis ratio				
Mean ± SD	1.30 ± 0.75	1.54 ± 1.51	1.26 ± 0.51	.966*
<1.0	43 (29.1)	7 (30.4)	36 (28.8)	.874†
≥1.0	105 (70.9)	16 (69.6)	89 (71.2)	
ALP	102.59 ± 92.54	130.22 ± 140.34	97.50 ± 80.56	.755*
Bilirubin	1.21 ± 2.36	1.49 ± 2.47	1.16 ± 2.35	.808*
Total protein	6.28 ± 0.79	6.21 ± 0.91	6.29 ± 0.77	.642§
Albumin	3.81 ± 0.62	**3.48 ± 0.74**	**3.87 ± 0.58**	**.005** §
Albumin-to-globulin ratio				
Mean ± SD	1.59 ± 0.38	**1.34 ± 0.38**	**1.64 ± 0.36**	**.002** *
<1.0	6 (4.1)	**4 (17.4**)	**2 (1.6**)	**.006** ‡
≥1.0	142 (95.9)	**19 (82.6**)	**123 (98.4**)	
BUN	14.08 ± 4.49	14.17 ± 6.32	14.06 ± 4.10	.451*
Creatinine	0.74 ± 0.23	0.68 ± 0.27	0.75 ± 0.22	.193§
GGT	99.65 ± 177.03	214.19 ± 325.03	79.60 ± 128.05	.051*
CRP	1.54 ± 3.14	3.88 ± 5.66	1.10 ± 2.18	.054*
PT INR	1.06 ± 0.11	1.08 ± 0.14	1.06 ± 0.11	.505*
CEA	204.36 ± 967.56	35.80 ± 79.94	234.51 ± 1047.78	.402*
CA 19-9				
Mean ± SD	10570.19 ± 28810.96	**12185.71 ± 30824.77**	**10296.60 ± 28579.21**	**.021** *
<39.0	99 (68.3)	**9 (42.9**)	**90 (72.6**)	**.007** †
≥39.0	46 (31.7)	**12 (57.1**)	**34 (27.4**)	
Pathology				
Adenocarcinoma (ADC)	133 (89.9)	20 (87.0)	113 (90.4)	.705‡
Non-ADC	15 (10.1)	3 (13.0)	12 (9.6)	
Pathological differentiation				
Well	64 (49.2)	**4 (19.0**)	**60 (55.0**)	**.003** §
Non-well	66 (50.8)	**17 (81.0**)	**49 (45.0**)	

Data are presented as mean ± SD or number (%), unless otherwise indicated. Bold values indicate statistical significance (*P* < .05).

The Shapiro–Wilk test was employed for test of normality assumption.

ALP = alkaline phosphate, ALT = aspartate aminotransferase, AST = aspartate aminotransferase, BMI = body mass index, CA 19-9 = carbohydrate antigen 19-9, CEA = carcinoembryonic antigen, CRP = C-reactive protein, DM = diabetes mellitus, GGT = gamma-glutamyl transpeptidase, HbA1C = hemoglobin A1C test, HT = hypertension, WBC = white blood cells.

* *P* values were derived from Mann–Whitney *U* test.

† *P* values were derived from chi-square test.

‡ *P* values were derived from Fisher exact test.

§ *P* values were derived from independent *t* test.

### 3.2. Risk factors associated with early recurrence

Based on the statistical analysis, the independent risk factors of early recurrence were age over 65 years (hazard ratio [HR]: 4.44, 95% confidence interval [CI]: 1.25–15.75, *P* = .021), glycated hemoglobin (HbA1c) level > 6.5% (HR: 5.00, 95% CI: 1.37–18.31, *P* = .015), surgical T stage > T3 (HR: 16.76, 95% CI: 5.76–48.73, *P* < .001), surgical N stage > N1 (HR: 5.82, 95% CI: 2.22–15.28, *P* < .001), moderate or poor pathological differentiation (HR: 5.20, 95% CI: 1.64–16.48, *P* = .005), reversed albumin–globulin (A/G) ratio (HR: 0.08, 95% CI: 0.01–0.45, *P* = .004), elevated gamma-glutamyl transpeptidase (GGT) level (HR: 1.00, 95% CI: 1.00–1.01, *P* = .007), high C-reactive protein (CRP) level (HR: 1.22, 95% CI: 1.08–1.38, *P* = .001), and high carbohydrate antigen (CA) 19-9 level (HR: 3.53, 95% CI: 1.36–9.13, *P* = .004) (Table 2).

**Table 2:** Univariate logistic regression analyses of early recurrence (N = 148).

Variable	Univariate analysis		
	OR	95% CI	*P*
Age (≥65 yr vs < 65 yr)	**4.44**	**(1.25–15.75**)	**.021**
Sex (male vs female)	0.86	(0.34–2.17)	.743
BMI (kg/m^2^) (≥25.0 vs < 25.0)	0.87	(0.32–2.39)	.791
Surgical T stage (T3 + T4 vs T1 + T2)	**16.76**	**(5.76–48.73**)	**<.001**
Surgical N stage (N1 + N2 vs N0)	**5.82**	**(2.22–15.28**)	**<.001**
Metabolic factors based on medical records			
HT (yes vs no)	0.41	(0.15–1.10)	.077
DM (yes vs no)	0.99	(0.31–3.18)	.981
Dyslipidemia (yes vs no)	1.29	(0.34–4.95)	.708
Inflammatory factors based on medical records			
Viral hepatitis (yes vs no)	0.00	(0.00)	.999
Liver cirrhosis (yes vs no)	0.59	(0.07–4.86)	.620
Biliary inflammation (cholecystolithiasis + cholecystitis vs no)	0.37	(0.13–1.05)	.061
Smoking (yes vs no)	1.02	(0.27–3.83)	.974
Alcohol (yes vs no)	0.76	(0.24–2.44)	.649
Cholesterol (≥200.0 vs < 200.0)	1.07	(0.33–3.48)	.912
HbA1C (≥6.5 vs < 6.5)	**5.00**	**(1.37–18.31**)	**.015**
WBC	0.99	(0.89–1.10)	.875
Neutrophil (%)	1.00	(0.97–1.02)	.781
Lymphocyte (%)	1.01	(0.97–1.04)	.669
Neutrophil-to-lymphocyte ratio (≥3.0 vs < 3.0)	1.11	(0.45–2.75)	.825
AST	1.00	(1.00–1.00)	.728
ALT	1.00	(1.00–1.01)	.653
De Ritis ratio	1.45	(0.87–2.41)	.155
De Ritis ratio (≥1.0 vs < 1.0)	0.92	(0.35–2.44)	.874
ALP	1.00	(1.00–1.01)	.139
Bilirubin	1.05	(0.90–1.23)	.549
Total protein	0.87	(0.49–1.54)	.639
Albumin	**0.34**	**(0.15–0.74**)	**.007**
Albumin-to-globulin ratio	**0.08**	**(0.02–0.33**)	**.001**
Albumin-to-globulin ratio (≥1.0 vs < 1.0)	**0.08**	**(0.01–0.45**)	**.004**
BUN	1.01	(0.91–1.11)	.918
Creatinine	0.23	(0.03–2.10)	.195
GGT	**1.00**	**(1.00–1.01**)	**.007**
CRP	**1.22**	**(1.08–1.38**)	**.001**
PT INR	4.58	(0.12–181.63)	.417
CEA	1.00	(1.00–1.00)	.442
CA 19-9 (≥39.0 vs < 39.0)	**3.53**	**(1.36–9.13**)	**.009**
Pathology (adenocarcinoma [ADC] vs non-ADC)	0.71	(0.18–2.74)	.616
Pathological differentiation (non-well vs well)	**5.20**	**(1.64–16.48**)	**.005**

The effect of independent variables on relapse-free survival (RFS) was analyzed using multivariable Cox regression, and statistically significant variables were included from the univariate analyses with 0.10 alpha level. Bold values indicate statistical significance (*P* < .05); values with *P* < .01 or < .001 are also indicated in bold.

ALP = alkaline phosphate, ALT = aspartate aminotransferase, AST = aspartate aminotransferase, BMI = body mass index, CA 19-9 = carbohydrate antigen 19-9, CEA = carcinoembryonic antigen, CRP = C-reactive protein, DM = diabetes mellitus, GGT = gamma-glutamyl transpeptidase, HbA1C = hemoglobin A1C test, HT = hypertension, WBC = white blood cells.

### 3.3. Risk factors associated with relapse-free-survival

According to the investigation, risk factors related to RFS were age over 65 years (HR: 3.82, 95% CI: 1.71–8.56, *P* = .001), HbA1c level > 6.5% (HR: 2.72, 95% CI: 1.31–5.66, *P* = .007), surgical T stage > T3 (HR: 5.60, 95% CI: 3.05–10.25, *P* < .001), surgical N stage > N1 (HR: 4.07, 95% CI: 2.25–7.36, *P* < .001), moderate or poor pathological differentiation (HR: 2.80, 95% CI: 1.46–5.37, *P* = .002), reversed A/G ratio (HR: 0.16, 95% CI: 0.06–0.40, *P* < .001), elevated GGT level (HR: 1.00, 95% CI: 1.00–1.00, *P* < .001), high CRP level (HR: 1.12, 95% CI: 1.04–1.21, *P* = .004), and high CA 19-9 = carbohydrate antigen 19-9 (CA 19-9) level (HR: 1.72, 95% CI: 0.94–3.17, *P* = .080) (Table 3).

**Table 3:** Univariate Cox proportional hazards regression analyses of relapse-free survival (N = 148).

Variable	Univariate analysis		
	HR	95% CI	*P*
Age (≥ 65 yr vs < 65 yr)	**3.82**	**(1.71–8.56**)	**.001**
Sex (male vs female)	**1.86**	**(1.04–3.32**)	**.036**
BMI (kg/m^2^) (≥ 25.0 vs < 25.0)	0.78	(0.39–1.53)	.463
Surgical T stage (T3 + T4 vs T1 + T2)	**5.60**	**(3.05–10.25**)	**<.001**
Surgical N stage (N1 + N2 vs N0)	**4.07**	**(2.25–7.36**)	**<.001**
Metabolic factors based on medical records			
HT (yes vs no)	0.57	(0.31–1.06)	.078
DM (yes vs no)	1.02	(0.48–2.19)	.954
Dyslipidemia (yes vs no)	1.16	(0.46–2.93)	.756
Inflammatory factors based on medical records			
Viral hepatitis (yes vs no)	1.95	(0.70–5.44)	.204
Liver cirrhosis (yes vs no)	1.20	(0.43–3.36)	.728
Biliary inflammation (cholecystolithiasis + cholecystitis vs no)	0.55	(0.29–1.05)	.070
Smoking (yes vs no)	1.91	(0.95–3.85)	.071
Alcohol (yes vs no)	1.20	(0.61–2.35)	.606
Cholesterol (≥ 200.0 vs < 200.0)	0.98	(0.46–2.11)	.962
HbA1C (≥ 6.5 vs <.6.5)	**2.72**	**(1.31–5.66**)	**.007**
WBC	0.99	(0.93–1.06)	.829
Neutrophil (%)	1.00	(0.98–1.01)	.691
Lymphocyte (%)	1.00	(0.98–1.03)	.767
Neutrophil-to-lymphocyte ratio (≥3.0 vs <3.0)	0.98	(0.54–1.75)	.939
AST	1.00	(1.00–1.00)	.964
ALT	1.00	(0.99–1.00)	.773
De Ritis ratio	1.48	(0.99–2.20)	.056
De Ritis ratio (≥ 1.0 vs <1.0)	0.95	(0.51–1.79)	.883
ALP	**1.00**	**(1.00–1.00**)	**.017**
Bilirubin	1.06	(0.98–1.15)	.129
Total protein	0.85	(0.58–1.24)	.395
Albumin	**0.39**	**(0.23–0.65**)	**<.001**
Albumin-to-globulin ratio	**0.13**	**(0.05–0.33**)	**<.001**
Albumin-to-globulin ratio (≥1.0 vs <1.0)	**0.16**	**(0.06–0.40**)	**<.001**
BUN	1.00	(0.93–1.07)	.951
Creatinine	0.98	(0.26–3.65)	.971
GGT	**1.00**	**(1.00–1.00**)	**<.001**
CRP	**1.12**	**(1.04–1.21**)	**.004**
PT INR	8.04	(0.76–85.38)	.084
CEA	1.00	(1.00–1.00)	.241
CA 19-9 (≥ 39.0 vs < 39.0)	**1.72**	**(0.94–3.17**)	**.080**
Pathology (adenocarcinoma [ADC] vs non-ADC)	1.31	(0.47–3.69)	.604
Pathological differentiation (non-well vs well)	**2.80**	**(1.46–5.37**)	**.002**

The effect of independent variables on relapse-free survival (RFS) was analyzed using Cox regression analysis, and statistically significant variables were included from univariate analyses with 0.10 alpha level. Bold values indicate statistical significance (*P* < .05); values with *P* < .01 or < .001 are also indicated in bold.

ALP = alkaline phosphate, ALT = aspartate aminotransferase, AST = aspartate aminotransferase, BMI = body mass index, CA 19-9 = carbohydrate antigen 19-9, CEA = carcinoembryonic antigen, CRP = C-reactive protein, DM = diabetes mellitus, GGT = gamma-glutamyl transpeptidase, HbA1C = hemoglobin A1C test, HT = hypertension, WBC = white blood cells.

## 4. Discussion

Biliary tract cancer is rare, accounting for 3% of all gastrointestinal cancers worldwide.^[12]^ When the epidemiology of biliary tract cancer is surveyed in detail, the incidence varies based on the geographical prevalence. While the incidence is low in Western countries,^[13]^ in endemic countries, including South Korea, the incidence is up to 40 times higher.^[14]^ The mortality rate of biliary tract cancer is extremely high,^[15]^ and according to a worldwide epidemiological study, South Korea showed the highest mortality rate (deaths per 100,000 person-years) of 11.64.^[16]^ Therefore, biliary tract cancer is a major cancer-related healthcare issue in South Korea.

Complete resection remains the only curative-intent therapy with surgical feasibility for patients with biliary tract cancer. However, most patients are initially diagnosed with unresectable or metastatic tumors. Moreover, biliary tract cancers have a high recurrence rate despite complete resection.^[17]^ When patients experience distant metastases, the 5-year overall survival rate drops to 5%, indicating an extremely poor prognosis.^[18]^ The aggressive disease course of biliary tract cancer necessitates the identification of risk factors strongly associated with early recurrence and RFS.

The established risk factors for biliary tract cancer are sorted into groups including primary hepatobiliary disease, toxic exposure, infections, and metabolic causes. Primary hepatobiliary diseases include primary sclerosing cholangitis and fibropolycystic liver diseases such as choledochal cysts; gallstone-related disorders such as cholelithiasis or cholecystitis; and chronic liver diseases related to viral hepatitis or liver cirrhosis. A social history of smoking or alcohol use is related to toxin exposure. Liver flukes, closely related to *C sinensis*, are a unique cause of infection. Metabolic factors such as diabetes and obesity are contributing risk factors.^[19]^ Geographical variation in biliary tract cancer is assumed to be correlated with different risk factors. In endemic East Asian regions, the major risk factors are parasitic *C sinensis* and hepatitis B and C viruses.^[20]^ However, in Western countries, primary sclerosing cholangitis or metabolic causes, including diabetes or obesity, are the dominant risk factors.^[21]^ Related to this geographical variation and risk factor correlation, intrahepatic cholangiocarcinoma associated with metabolic syndrome is prevalent in Western countries,^[22]^ while extrahepatic cholangiocarcinoma is prevalent in endemic areas, including South Korea.^[23]^

Biliary tract cancers are classified as intrahepatic cholangiocarcinoma, perihilar cholangiocarcinoma, extrahepatic cholangiocarcinoma, and GB cancer based on anatomical differences.^[3]^ According to the Korean National Cancer Center Surveillance Data, GB cancers constitute most cases of biliary tract cancers, with approximately 2600 cases annually.^[24]^ In curative intent, R0 resection with lymphadenectomy is the best curative treatment for operable GB. However, the complete surgical resection rate is up to 30%, and approximately half of the patients have a chance of recurrence despite complete resection.^[25]^ It is reasonable to presume that the risk of recurrence, despite after R0 resection, might be related to the predisposing risk factors for GB cancer.

Demographic characteristics, medical history, and basic laboratory results were analyzed in all patients enrolled. Most of these parameters analyzed were commonly assessed and easily obtained during outpatient or inpatient care. Our team assessed these factors to enhance the clinical applicability of our findings and support their potential use in early risk screening. Furthermore, our study attempted to derive more predictive markers from standard laboratory values. For example, the A/G ratio^[26]^ and De Ritis (aspartate transaminase/alanine transaminase) ratio,^[27]^ which are estimated as a prognostic inflammation indicator in some malignancies, were investigated in this study.

According to our analysis, an advanced pathological stage strongly predicted early recurrence and poor RFS. All the enrolled patients underwent R0 resection, which is theoretically a curative treatment. However, patients with surgical T stage > T3, surgical N stage > N1, and moderate or poor pathological differentiation showed poor survival outcomes. Previous studies have implied that the prevalence of postoperative relapse in GB cancer is approximately 25% because of early metastasis.^[28]^ Presumably, an advanced surgical stage reflects a higher microscopic residual disease burden, and poor differentiation is correlated with a higher risk of aggressive local infiltration. Therefore, adjuvant chemotherapy after an R0 resection may reduce the risk of recurrence.^[29]^

High levels of inflammatory markers have been associated with poor surgical outcomes. Previous studies have shown that several inflammatory markers reflect systemic inflammatory response and can be used as predictive biomarkers of postoperative morbidity and mortality in patients with gastrointestinal cancer.^[30]^ In our study, a reversed A/G ratio, elevated GGT levels, high CRP levels, and high CA 19-9 levels were associated with reduced RFS. Albumin and globulin are major serum proteins that represent systemic inflammation.^[31]^ GGT is a marker of hepatic injury, and its elevation is associated with an increased risk of chronic diseases.^[32]^ Although mostly used as an acute-phase inflammatory marker, a moderate increase in CRP levels has been observed in chronic inflammatory states, including malignancy.^[33]^ CA 19-9 is the most valid serum tumor marker for PC diagnosis and monitoring of pancreatic cancer. CA 19-9 is synthesized by the pancreatic and biliary ductal cells, and its serum level is elevated in the presence of a malignancy, especially of gastrointestinal origin.^[34]^ It is assumed that these inflammatory markers may reflect the tumor burden of patients. Although irrelevant when analyzed separately, these markers may play a role in predicting survival outcomes if interpreted in combination. However, further evaluation of this concept is necessary.

Based on our report, a high HbA1c level was also associated with early recurrence. Previous studies have suggested a correlation between diabetes mellitus and cancer. According to 1 meta-analysis, the risk of intrahepatic and extrahepatic cholangiocarcinoma increased in patients with diabetes compared to that in patients without diabetes.^[35]^ Studies imply that metabolic factors, including obesity, dyslipidemia, hypertension, and diabetes, are more closely related to intrahepatic cholangiocarcinoma.^[36]^ This tendency is also consistent with the geographical variation in biliary tract cancer. Based on our findings, it can be inferred that an increased incidence of diabetes may aggravate the risk of GB cancer. Early screening and intensive management of blood glucose control may help improve RFS. Based on these findings, we are designing a subsequent study to explore whether interventions such as weight reduction and improvements in cardiopulmonary fitness through rehabilitation programs could help prevent GB cancer recurrence after surgery.

There are some limitations in this study. During data analysis, including waist circumference as a predictive factor was considered. Waist circumference is a well-known contributing factor of metabolic syndrome, which has recently emerged as a strong predictor of cancer incidence.^[37]^ However, this parameter was not included in the final analysis due to some reasons. First, waist circumference was not consistently recorded in the medical records of the enrolled patients. It was concerned that that including a variable with a high proportion of missing data would significantly reduce the statistical power of the study. Although we considered estimating abdominal circumference using preoperative CT scans as an alternative, concerns about reproducibility and measurement consistency limited its feasibility. Nonetheless, we recognize the potential value of this parameter and intend to explore it further in future studies.

Another limitation is that most of the patients included in our study were treated prior to the publication of the BILCAP trial,^[27]^ which established capecitabine as the standard adjuvant chemotherapy regimen for biliary tract cancers in many clinical guidelines. Most patients with R1 or R2 resections in our cohort dataset did not receive guideline-based adjuvant treatment. This included tegafur-uracil monotherapy, 5-fluorouracil–cisplatin combinations, or even radiation therapy alone. It can be assumed that these regimens generally offer lower efficacy and may be associated with higher toxicity compared to the capecitabine-alone regimen. Moreover, it was concerned that including these patients in the analysis could compromise the objectivity and interpretability of study outcomes. For these reasons, whether patients received adjuvant chemotherapy was not included as a variable in this study. Despite that, our team expects that future research involving patients treated with the current standard of adjuvant therapy will allow for more consistent and meaningful evaluation of recurrence prevention.

## 5. Conclusions

This study aimed to investigate risk factors strongly related to recurrence after R0 resection of GB cancer. In total, 148 patients with GB cancer who underwent R0 resection were analyzed with statistical tools to identify variables related to prognosis. Factors reflecting high tumor burden, such as advanced pathological stage and high inflammatory marker levels, were related to poor surgical outcomes. Moreover, high HbA1c level was associated with poor prognosis. It is assumed that modifying these predictors may contribute to improve prognosis. Active screening for diagnosis at an earlier stage, reduction in inflammatory conditions, and management of diabetes may reduce early recurrence and enhance RFS after R0 resection of GB cancer.

## Author contributions

**Conceptualization:** Dong Uk Kim.

**Data curation:** Dong Uk Kim.

**Formal analysis:** Young Mi Seol.

**Funding acquisition:** Dong Uk Kim.

**Investigation:** Young Mi Seol.

**Methodology:** Young Mi Seol.

**Project administration:** Dong Uk Kim.

**Resources:** Sang Hun Lee.

**Software:** Sang Hun Lee.

**Supervision:** Young Mi Seol.

**Validation:** Sang Hun Lee.

**Visualization:** Sang Hun Lee.

**Writing – original draft:** Sang Hun Lee.

**Writing – review & editing:** Sang Hun Lee.
